# Early Symblepharon Associated With Periocular Cosmetic Use in a Young Woman With Chronic Dry Eye Disease: A Case Report

**DOI:** 10.7759/cureus.108366

**Published:** 2026-05-06

**Authors:** Kristo Hadi Audric Sugiaman, Stefenie Stefenie, Gede Pardianto

**Affiliations:** 1 Department of Ophthalmology and Vision Sciences, Universitas Prima Indonesia, Medan, IDN; 2 Department of Ophthalmology, Prima Vision Eye Hospital, Medan, IDN

**Keywords:** conjunctival adhesion, digital screen exposure, dry eye disease (ded), evaporative dry eye, eye cosmetic, meibomian gland dysfunction (mgd), ocular surface inflammation, periocular cosmetic, symblepharon, tear film instability

## Abstract

Chronic dry eye disease (DED) is increasingly observed in younger individuals and is often associated with modifiable lifestyle factors. We report the case of a 27-year-old woman presenting with persistent ocular irritation, redness, and foreign-body sensation unresponsive to prior topical therapy. Clinical evaluation revealed mild conjunctival hyperemia and a localized inferonasal adhesion between the bulbar and tarsal conjunctiva in the right eye, consistent with early symblepharon formation. The findings were suggestive of evaporative DED exacerbated by habitual periocular cosmetic use and prolonged digital screen exposure.

Management included discontinuation of cosmetic products, initiation of preservative-free lubricants, topical anti-inflammatory therapy, and implementation of eyelid hygiene and environmental modifications. At two-week follow-up, the patient demonstrated marked symptomatic improvement, with reduced ocular discomfort and no progression of the conjunctival adhesion.

This case points out the possible role of chronic cosmetic exposure as an underrecognized contributor to ocular surface inflammation, tear film instability, and early conjunctival scarring. Early identification of modifiable risk factors and timely intervention are essential to prevent progression and preserve long-term ocular surface integrity.

## Introduction

Dry eye disease (DED) is a multifactorial disorder of the ocular surface characterized by discomfort, visual disturbances, and tear-film instability, with potential damage to the ocular epithelium [1]. Recent epidemiological trends indicate a rising prevalence of DED among younger individuals, largely attributed to lifestyle and environmental factors, including prolonged digital device use and increasing cosmetic application [2].

Periocular cosmetic products have been increasingly recognized as contributors to ocular surface dysfunction. The Tear Film and Ocular Surface Society (TFOS) Lifestyle Workshop (2023) highlighted that cosmetic use may disrupt the lipid layer of the tear film and impair meibomian gland function (the oil-producing glands in the eyelids), thereby exacerbating evaporative DED, a condition where the tear film dries too quickly due to a deficient lipid layer [3]. Migration of eyeliner and mascara particles into the tear film has been shown to increase ocular surface staining and reduce tear stability [4,5]. In addition, preservatives and pigments contained in cosmetic products may induce local inflammation and epithelial toxicity [6]. Disruption of the ocular surface and tear film as an integrated functional unit plays a central role in the pathogenesis of DED [7].

Symblepharon is defined as an abnormal adhesion between the eyelid and the eyeball conjunctiva and is typically associated with severe ocular surface injury, such as chemical burns, trauma, or cicatrizing conjunctival disorders. However, chronic low-grade inflammation and mechanical irritation may also contribute to its development. Despite growing awareness of lifestyle-related ocular surface disease, the association between habitual cosmetic use and early conjunctival adhesion is rarely documented in current literature, as most reports focus on biochemical tear film disruption rather than structural cicatricial changes. While the inflammatory effects of cosmetics on the tear film are increasingly recognized, their progression to permanent structural alterations like symblepharon represents a far more severe and rare clinical manifestation that is seldom documented. This report describes a case of chronic DED with early symblepharon formation associated with long-term periocular cosmetic use in a young woman, highlighting the potential role of modifiable lifestyle factors in the development of ocular surface pathology.

## Case presentation

A 27-year-old woman presented with acute onset of pruritus in the right eye for one day, superimposed on a two-month history of intermittent ocular burning, dryness, and mild conjunctival redness. The symptoms were described as a persistent foreign-body sensation with fluctuating discomfort, without photophobia, significant pain, or visual impairment. The patient reported prior self-medication with topical antibiotic and corticosteroid combinations (tobramycin/dexamethasone and neomycin/polymyxin B/dexamethasone) for several weeks without symptomatic improvement.

The patient admitted to daily use of eyeliner and mascara, often incompletely removed using only facial cleansers. She worked in an air-conditioned environment and reported prolonged daily exposure to digital screens. There was no history of contact lens use, ocular trauma, prior ocular surgery, use of systemic medications (e.g., anti-diabetics or glaucoma drops), or symptoms suggestive of systemic cicatrizing diseases such as oral or skin lesions.

**Figure 1:**
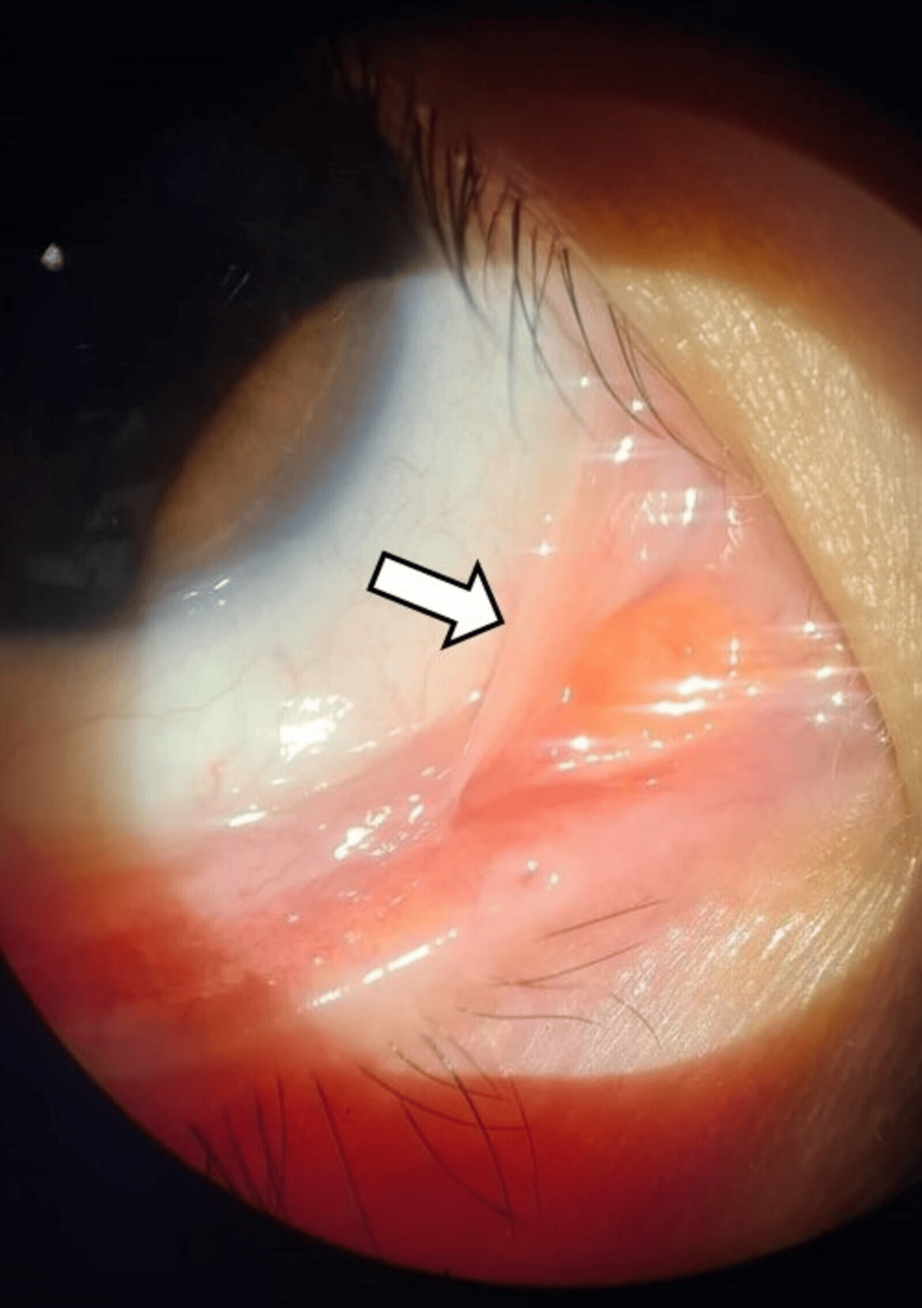
Slit-lamp photograph of the right eye The white arrowhead points to the localized focal bridge of tissue (symblepharon) between the bulbar and tarsal conjunctiva in the inferonasal region with mild surrounding conjunctival hyperemia.

On examination, the best-corrected visual acuity was 6/7.5 in both eyes with mild myopic correction (−0.75 diopters). Intraocular pressure was 12 mmHg in both eyes. Slit-lamp examination of the right eye revealed mild diffuse conjunctival hyperemia and a reduced tear film break-up time (estimated at <5 seconds) with diffuse punctate epithelial erosions on fluorescein staining. A focal, non-restrictive inferonasal adhesion between the bulbar and tarsal conjunctiva, approximately 2 mm in width, was identified, consistent with early symblepharon formation (Figure 1). The adhesion did not limit extraocular movements.

The cornea, anterior chamber, iris, and lens were unremarkable. Pupils were equal and reactive to light. Examination of the left eye was within normal limits. A diagnosis of evaporative DED with early symblepharon in the right eye was established. Management included preservative-free hydroxypropyl methylcellulose (HPMC) eye drops administered four times daily and topical fluorometholone 0.1% twice daily. The patient was advised to discontinue all periocular cosmetic use, avoid eye rubbing, perform warm compresses twice daily, maintain eyelid hygiene, and reduce exposure to air conditioning and prolonged screen use.

At the two-week follow-up, the patient reported marked symptomatic improvement, with decreased ocular discomfort and redness. The conjunctival adhesion remained stable without progression, and ocular surface staining had significantly diminished. Table 1 features a summary of the patient's clinical presentation, diagnosis, and management.

**Table 1:** Summary of clinical presentation, diagnosis, and management DED: Dry eye disease

Category	Description
Chief complaint	Acute pruritus, chronic burning, and foreign body sensation (OD)
Key risk factors	Habitual eyeliner/mascara use, incomplete removal, prolonged screen time and exposure to air-conditioning
Objective findings	Focal inferonasal symblepharon (2mm), conjunctival hyperemia, tear film break-up time <5 seconds, punctate epithelial erosions
Diagnosis	Evaporative DED with early localized symblepharon (OD)
Management	Discontinuation of cosmetics, preservative-free lubricants, topical fluorometholone, and eyelid hygiene
Outcome (two weeks)	Significant symptomatic relief, stable adhesion, and resolved surface staining

## Discussion

Dry eye disease is a multifactorial disorder in which tear film instability, ocular surface inflammation, and neurosensory abnormalities interact in a self-perpetuating cycle. While traditionally more prevalent in older populations, recent epidemiological trends indicate a growing burden among younger individuals, largely driven by modifiable environmental and behavioral factors. The present case highlights habitual periocular cosmetic use as an underrecognized yet clinically relevant contributor to ocular surface dysfunction in this demographic.

Periocular cosmetic products may adversely affect the ocular surface through both mechanical and biochemical mechanisms. Migration of eyeliner and mascara particles into the tear film and along the eyelid margin has been shown to disrupt the lipid layer and promote tear evaporation. In addition, deposition of cosmetic debris at the meibomian gland orifices (the openings of the oil glands in the eyelids) may impair glandular function, contributing to meibomian gland dysfunction (MGD), a key driver of evaporative DED (where tears evaporate too quickly) [8,9]. These mechanical effects are compounded by potential chemical toxicity from preservatives, pigments, and other additives, which may induce epithelial stress and subclinical inflammation, thereby destabilizing tear film homeostasis [10].

The impact of cosmetic use in this case should also be interpreted within a broader context of environmental and lifestyle exposures. Prolonged digital screen use is associated with reduced blink rate and incomplete blinking, both of which exacerbate tear film instability and evaporative loss. Similarly, chronic exposure to air-conditioned environments has been implicated in increased tear evaporation and ocular surface desiccation. The coexistence of these factors in this patient likely created a synergistic effect, amplifying ocular surface stress and symptom severity [11].

A particularly notable feature of this case is the presence of early, localized symblepharon (an abnormal adhesion between the eyelid and eyeball conjunctiva). Symblepharon is classically associated with severe ocular surface injury, including chemical burns, Stevens-Johnson syndrome, or ocular cicatricial pemphigoid. However, emerging evidence suggests that chronic low-grade inflammation, when combined with repetitive mechanical microtrauma, may also induce localized fibroinflammatory changes leading to early conjunctival adhesion. From a clinical standpoint, when such adhesions are observed, a systematic evidence-based approach is vital. While lifestyle factors like cosmetic use should be investigated, clinicians must prioritize ruling out systemic or drug-induced causes [12].

In this case, the temporal relationship between long-term cosmetic use and the gradual onset of symptoms, culminating in localized adhesion, suggests a cumulative inflammatory effect. When evaluating such presentations, a robust differential diagnosis is essential. We considered allergic and toxic conjunctivitis; however, the absence of a prominent follicular or papillary response and the specific structural finding of symblepharon, which is atypical for simple allergic reactions, favored a diagnosis of chronic low-grade inflammation. Furthermore, the lack of systemic mucosal involvement and the non-progressive nature of the lesion after the withdrawal of cosmetics helped differentiate this from early-stage ocular cicatricial pemphigoid (OCP) or drug-induced pseudopemphigoid.

This case serves as a critical reminder for clinicians to meticulously inspect the conjunctival fornices for subtle structural changes, such as early adhesions or symblepharon, in patients presenting with chronic or recurrent low-grade conjunctivitis. From a clinical standpoint, when such adhesions are observed, a systematic evidence-based algorithmic approach is essential for identifying the underlying etiology and differentiating between lifestyle-induced changes and progressive systemic diseases [7]. While lifestyle factors like cosmetic use should be investigated, clinicians must prioritize ruling out systemic or drug-induced causes.

From a pathophysiological perspective, persistent inflammation may activate fibroblasts and promote extracellular matrix remodeling. This mechanism provides a plausible explanation for the early development of conjunctival adhesion in the present case. However, we acknowledge several significant limitations in our diagnostic approach. Without a conjunctival biopsy, the gold standard for ruling out OCP, and a long-term follow-up period to confirm the lack of disease progression after the withdrawal of the offending agent, the causative role of periocular cosmetics remains plausible but not definitive [10,13].

Importantly, the clinical course observed in this patient underscores the potential reversibility of early ocular surface dysfunction when modifiable risk factors are promptly addressed. Discontinuation of cosmetics, combined with therapy, resulted in rapid symptomatic improvement. Objectively, the resolution of punctate epithelial erosions and the stabilization of the conjunctival adhesion at the two-week follow-up confirm the effectiveness of targeting these modifiable factors.

From a clinical standpoint, this case emphasizes the need for comprehensive history-taking that includes detailed inquiry into cosmetic practices, particularly in younger patients presenting with chronic ocular irritation. Cosmetic use is often underreported and may not be routinely considered during ophthalmic evaluation. Patient education regarding safe cosmetic application, including avoidance of 'tightlining' (applying eyeliner inside the lash line), proper removal techniques, and minimizing product accumulation along the eyelid margin, should be incorporated into routine clinical care.

To our knowledge, reports describing early symblepharon associated with habitual cosmetic use in otherwise healthy young individuals remain limited. This case, therefore, expands the current understanding of behaviorally induced ocular surface disease and suggests that chronic cosmetic exposure, particularly when combined with environmental stressors, may contribute not only to tear film instability and inflammation but also to early structural alterations of the conjunctiva.

We acknowledge that without a conjunctival biopsy and long-term follow-up, systemic cicatrizing diseases such as OCP cannot be definitively ruled out. However, the absence of systemic symptoms and the localized nature of the adhesion following chronic cosmetic exposure suggest a plausible link that warrants further investigation.

## Conclusions

Chronic periocular cosmetic use is a plausible contributor to tear film instability, ocular surface inflammation, and potential early conjunctival adhesion. This case highlights the importance of recognizing modifiable lifestyle factors, alongside environmental stressors and prior medication use, in younger patients presenting with persistent ocular irritation. While the observational nature of this report precludes a definitive causal establishment, early identification, patient education on cosmetic hygiene, and timely intervention remain essential to prevent potential progression and preserve long-term ocular surface integrity.
